# Validation of EGCRISC for Chronic Hepatitis C Infection Screening and Risk Assessment in the Egyptian Population

**DOI:** 10.1371/journal.pone.0168649

**Published:** 2016-12-21

**Authors:** Engy Mohamed El-Ghitany, Azza Galal Farghaly, Shehata Farag, Ekram Wassim Abd El-Wahab

**Affiliations:** 1 Tropical Health Department, High Institute of Public Health, Alexandria University, Egypt; 2 Biostatistics Department, High Institute of Public Health, Alexandria University, Egypt; Chang Gung Memorial Hospital Kaohsiung Branch, TAIWAN

## Abstract

Chronic HCV infection, a highly endemic disease in Egypt, is usually asymptomatic for decades after infection. Prediction questionnaire tool was proofed to be a valuable, feasible and efficient instrument for the screening of several diseases. We previously developed an Egyptian HCV risk screening tool (EGCRISC). This study aims to validate/modify EGCRISC. A cross-sectional study testing 4579 individuals by EGCRISC as well as ELISA/PCR was performed. The sample was a stratified cluster sampling from urban and rural areas in Upper and Lower Egypt using a proportional allocation technique. The degree of agreement and positive and negative posttest probabilities were calculated. ROC curve was done and the cutoff points were customized for best performance. The total score was further classified into three levels according to the risk load. The mean age of the participants was 41.1±12.2 in whom HCV prevalence was 8.6%. EGCRISC, particularly after modifying the cutoff points, has a good discriminating ability. The degree of agreement was at least 68.1% and the positive posttest probability ranged from 5% to 37.2% whereas the negative posttest probability was in the range 1% to 17%. We conclude that EGCRISC is a valid tool that can potentially screen for HCV infection risk in Egypt and could diminish the demand for mass serologic screening in those apparently at minimal risk. Extensive use of electronic and self- or interviewer-administered risk-based screening strategy may simplify and promote overall screening and detection of HCV dissimilar communities.

## Introduction

Early detection of chronic HCV infection and eventually treatment and lifestyle/ behavioral changes cannot only prevent sequelae such as cirrhosis, end-stage liver disease or HCC, but also interrupts infection transmission [1].

HCV is arguably the major public health challenge facing Egypt today. The virus shows evidence of continuous transmission in health care settings as well as within households [2]. Due to the absence of vaccines and drugs for post-exposure prophylaxis, precautionary measures preventing future spread is the cornerstone for prevention [3].

Because of the asymptomatic nature of HCV infection before diseases progression, many HCV infected individuals are not aware of their condition and therefore do not seek help or perceive a need to screen for HCV infection. As a result, a potentially large number of infected individuals remain unidentified or are identified late [3]. A major barrier to seeking HCV treatment is unawareness of HCV seropositivity [4].

People identified to be HCV infected benefit from counseling, risky behavior modification, HAV or HBV vaccinations, alcohol cessation and other interventions including the recently released effective antiviral treatment [5].

To control the epidemic in Egypt, extensive efforts should be directed towards identifying apparently healthy individuals with HCV infection. Risk calculation approaches have been widely applied in public health actions and clinical care and have even been accepted as preliminary diagnosis for some diseases [6].

The United States Preventive Services Task Force (USPSTF) concluded in 2004 that screening high-risk population would be more efficient strategy than screening average–risk population [7]. With increasing recognition of the clinical and public health benefit of early detection, a simple self-administered tool may provide means to identify infected individuals [8–10].

Few studies have evaluated screening tools for estimating risk for HCV infection to support efficient screening of the hidden population of HCV–infected individuals [11, 12]. Further research is needed to understand the effects of different strategies on clinical outcomes and to customize the tool to the target population. Accordingly, we -in a previous study [13]- developed a short version risk assessment tool for HCV infection screening in Egypt (EGCRISC). The present large scale cross-sectional study is aiming to validate and modify -if needed- the EGCRISC tool to be more effective in identifying those at increased risk of HCV infection in the Egyptian setting, a step in a road to apply this tool in the primary care settings and as an internet-based screening program.

## Methods

### Development of the prediction model

The risk assessment tool abstracted from the first phase [13] was developed through a multivariate model of independent predictors of HCV seropositivity, that included the significant factors detected in the bivariate analysis among two age strata (<45 and >45 years) for each gender. Variables were ranked by their magnitude of risk [(Odds Ratio (OR)], with an overall score represented by the simple arithmetic sum of the nearest integral values. “Table 1” summarizes the 17 overlapping predictors, ranging from 8 to 13 in each of the four stratified groups. The OR for each factor assigned its score, giving a different total score for each stratum. The cut-off value for each group was estimated using ROC curve analysis, based on Youden index criterion, to specify the discriminating point of the highest sensitivity and specificity.

**Table 1:** Summary of EGCRISC strata, factors, scores and cut-off points.

Risk Factor	Score			
	Male <45 yrs	Male >45 yrs	Female <45 yrs	Female >45 yrs
Blood/blood products transfusion	**9**	**8**	**4**	**2**
Rural Residence	**8**	**15**	**18**	**7**
Fatigue	**7**	**2**	**3**	**2**
History of jaundice	**6**	**3**	**4**	**1**
History of PAT	**2**	**3**	**9**	**6**
Incarceration	**2**	**4**	**-**	**-**
Unsafe rout of sex	**2**	**-**	**-**	**-**
Contact with jaundice patient	**2**	**-**	**-**	**-**
Use of barber or beautician tools	**2**	**-**	**-**	**3**
Substance abuse	**2**	**3**	**-**	**-**
Living abroad	**2**	**2**	**-**	**-**
Hospitalization	**2**	**-**	**3**	**-**
Needle prick	**2**	**2**	**-**	**2**
History of invasive procedures	**-**	**2**	**3**	**-**
Menses during intercourse	**-**	**-**	**4**	**-**
Blood sample	**-**	**-**	**2**	**-**
Labour and delivery at home	**-**	**-**	**-**	**2**
Total	**47**	**44**	**50**	**25**
Cut-off value	**11**	**8**	**11**	**7**

### Sample size

A sample of 4100 persons are required to estimate expected agreement with phase I scoring for predicting HCV infection status to be on average 70% with a tolerated error margin of 2%, confidence level of 95% and design effect = 2.

### Sampling technique and methods of selection

During this validation phase, the tool was applied to a stratified cluster sampling from urban and rural areas using a proportional allocation technique in governorates representing upper and lower Egypt considering a rural to urban ratio of 1.3:1 according to the latest national estimates [14]. In each governorate, a number of districts were selected at random where participants older than 15 years were voluntarily recruited.

### Laboratory investigations

According to the pre-specified cutoff points for males and females in both age groups (above and below 45 years), the cross sectional sample individuals were classified to potentially HCV infected and potentially non-HCV infected. The actual HCV status was determined using commercial 3^rd^ generation ELISA kits (DIALAB^®^, Austria). Confirmation of ELISA results was done using a kit from a different supplier (DiaSorin Murex^®^, version 4.0, Italy). Quantitative real time PCR was done for ELISA positive subjects to test for HCV-RNA. A cross validation between the predicted status by EGCRISC score and the actual status was done by calculating classification accuracy rate and the degree of agreement between both tests. A new cutoff points for EGCRISC score system was derived using c statistic of ROC curve to estimate the distance between the extracted point and the previously determined one by the case control phase as a crude measure of validity for the old score.

### Zones

Being at risk of having HCV based on a total score of the risk factors was categorized into zones; low risk (green zone), moderate risk (yellow zone) and high risk (red zone) using cluster analysis depending on the very large sample size and including all scored factors for discriminating cases into 3 groups using K-Means clustering technique, then and after identifying the extracted groups (clusters) of cases, the upper and lower limit for the overall score was identified as borders for each area which showed good concordance with the actual binary classification of persons.

### Ethical statement

The present study was approved by the ethics committee and institutional review board of the High Institute of Public Health, Alexandria University (Egypt). The research conformed to the ethical guidelines of the Declaration of Helsinki (2013) and the International Conference on Harmonization Guidelines for Good Clinical Practice. An informed written consent was signed by all participants invited after elaborating on the study aim and concerns. Data sheets were coded to ensure anonymity and confidentiality of participants’ data.

## Results

The study comprised a total of 4579 participants (55.7% males, 44.3% females) recruited from different urban and rural areas in Egypt “Table 2”. The mean age was 41.1±12.2. Other sociodemographic characteristics and a list of the studied HCV risk factors are detailed in “Table 3”. Stratified analysis of HCV risk factors by age and gender is displayed in (S1 Table).

**Table 2:** The studied cases according to the their demographics and HCV status in the study settings.

Item	Setting							
	Kafr Sheikh		Damanhur		Alexandria		Luxor	
	n = 1403		n = 103		n = 2036		n = 1037	
	No	%	No	%	No	%	No	%
**Gender**								
Female	730	52.0	31	30.1	679	33.3	590	56.9
Male	673	48.0	72	69.9	1357	66.7	447	43.1
**Age**								
<45	707	50.4	53	51.5	1329	65.3	597	57.6
45+	696	49.6	50	48.5	707	34.7	440	42.4
**Mean ± SD**	42.5 ± 15.4		42.7 ± 8.6		39.8± 11.2		41.3 ± 13.9	
**Anti-HCV ELISA**								
Negative	1205	85.9	98	95.1	1932	94.9	950	91.6
Positive	198	14.1	5	4.9	104	5.1	87	8.4
**PCR**								
Negative	1231	87.7	98	95.1	1955	96.0	965	93.1
Positive	172	86.9^(12.3)*	5	100.0^(4.9)*	81	77.9^(4.0)*	72	82.8^(6.9)*

^percentage from the ELISA positive subjects.

*percentage from the total.

**Table 3:** Sociodemographics and HCV risk factors including those of EGCRISC.

Factor		Anti-HCV ELISA				OR (95% CI)
		Negative		Positive		
		No	%	No	%	
**Residence**	Urban	1834	95.5	87	4.5	**2.8 (2.2–3.5)**
	Rural	2351	88.4	307	11.6	
**Education**	Illiterate /read & write	1440	84.7	261	15.3	**4.4 (2.9–6.7)**
	Basic	699	95.1	36	4.9	1.3 (0.74–2.1)
	Secondary	1437	95.2	72	4.8	1.2 (0.76–1.9)
	University / more	609	96.1	25	3.9	®
	Single	465	98.9	5	1.1	®
**Marital status**	Married	3394	91	336	9	**9.2 (3.8–22.4)**
	Divorced/widow	326	86	53	14	**15.1 (5.9–38.2)**
	Not working	1519	91.1	148	8.9	ref
**Job nature**	Low risk	2102	92.4	174	7.6	0.85 (0.67–1.1)
	High risk	564	88.7	72	11.3	1.3 (0.97–1.8)
**Working abroad**	No	3615	91.9	318	8.1	®
	Yes	570	88.2	76	11.8	**1.5 (1.2–1.9)**
**Tattooing**	No	3878	91.3	370	8.7	0.82 (0.53–1.3)
	Yes	307	92.7	24	7.3	
**Ear / body piercing**	No	2308	90.8	234	9.2	0.84 (0.68–1.1)
	Yes	1877	92.1	160	7.9	
**Shared instruments**	No	904	90	101	10	0.80 (0.63–1.1)
	Yes	3281	91.8	293	8.2	
**Use barber tools**	No	1026	88.4	134	11.6	**0.63 (0.51–0.78)**
	Yes	3159	92.4	260	7.6	
**Pierced with blood contaminated tool**	No	3851	91.5	357	8.5	1.2 (0.83–1.7)
	Yes	334	90	37	10	
**Bitten by animal**	No	3386	91.4	317	8.6	1.0 (0.79–1.3)
	Yes	799	91.2	77	8.8	
**Exposed to blood**	No	3305	91.6	305	8.4	1.1 (0.86–1.4)
	Yes	880	90.8	89	9.2	
**Blood / blood products transfusion**	No	3858	92.2	326	7.8	**2.5 (1.9–3.2)**
	Yes	327	82.8	68	17.2	
**History of jaundice**	No	3814	91.5	353	8.5	**1.2 (0.99–1.7)**
	Yes	371	90	41	10	
**Family member with hepatic disease**	No	3503	91.5	326	8.5	1.1 (0.82–1.4)
	Yes	682	90.9	68	9.1	
	No	591	93.2	43	6.8	®
**Blood sampling**	<10 years	2757	92.2	234	7.8	1.2 (0.83–1.6)
	>10 years	837	87.7	117	12.3	1.9 (0.89–2.7)
**Previous hospitalization**	No	1914	91.2	185	8.8	0.95 (0.77–1.2)
	Yes	2271	91.6	209	8.4	
**Bilharziasis**	No	2886	96.1	116	3.9	**5.3 (4.2–6.7)**
	Yes	1299	82.4	278	17.6	
**Genital ulcers**	No	3236	92	281	8	1.4 (1.0–1.7)
	Yes	949	89.4	113	10.6	
**Circumcision**	No	1061	96.6	37	3.4	**3.3 (2.3–4.6)**
	Yes	3124	89.7	357	10.3	
**Tarter injections for Bilharziasis**	No	3634	95.4	177	4.6	**8.1 (6.5–10.1)**
	Yes	551	71.7	217	28.3	
**History of oral ulcers**	No	2749	92	239	8	1.2 (1.0–1.5)
	Yes	1436	90.3	155	9.7	
**Invasive procedure**	No	3146	92.3	261	7.7	**1.5 (1.2–1.9)**
	Yes	1039	88.7	133	11.3	
**Acupuncture**	No	4075	91.3	389	8.7	0.47 (0.19–1.2)
	Yes	110	95.7	5	4.3	
**History of incarceration**	No	4098	91.4	385	8.6	1.1 (0.55–2.2)
	Yes	87	90.6	9	9.4	
**Opioid injection**	No	4183	91.4	393	8.6	5.3 (0.48–58.2)
	Yes	2	66.7	1	33.3	
**Drink alcohol**	No	3998	91.6	368	8.4	1.5 (0.98–2.3)
	Yes	187	87.8	26	12.2	
**Waterpipe smoking (Shisha)**	No	3423	91.7	311	8.3	1.1 (0.93–1.5)
	Yes	762	90.2	83	9.8	
**Previous stay at camps/hostels**	No	2880	92.1	247	7.9	1.3 (1.0–1.6)
	Yes	1305	89.9	147	10.1	
**Partner with STIs**	No	4021	91.3	381	8.7	0.84 (0.47–1.5)
	Yes	164	92.7	13	7.3	
**Partner infected with HCV**	No	4050	91.6	371	8.4	**1.8 (1.2–2.9)**
	Yes	135	85.4	23	14.6	
**Unexplained fatigue in last 6 months**	No	2889	93.3	207	6.7	**2.0 (1.6–2.5)**
	Yes	1296	87.4	187	12.6	

® Reference category

In the studied population, there was an 8.6% prevalence of HCV antibodies with HCV viremia found in 83.8% of them (7.2%).

**Fig 1:**
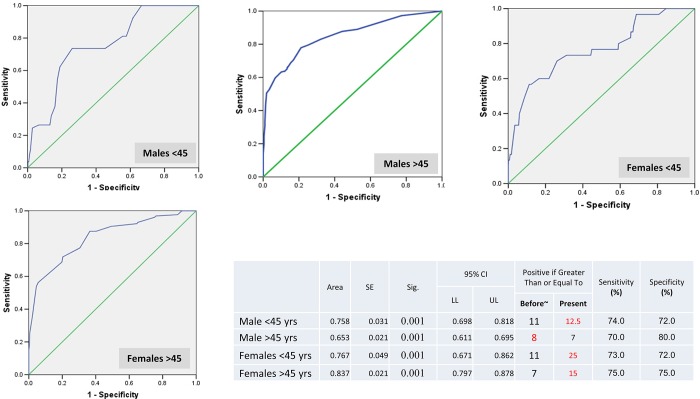
The scoring system for the selected risk factors. ROC curve analysis for the best cutoff point discriminating HCV positive and HCV negative status. Old cut off value was included in the table. Cutoff values respected in our prediction model are displayed in a red color font. ~ old cut off value

The EGCRISC has an average discriminating ability for persons' HCV status as the area under curve (AUC) ranged from 0.65 for older males (above 45 years) to 0.84 for older females. Based on ROC curve analysis, the best cutoff point discriminating HCV positive and HCV negative cases for each gender in the two age strata are displayed in (Fig 1). As for males above 45, we respected the use of the best cutoff point of phase I model (8 *vs* 7) since it had higher reported sensitivity (70% *vs* 66%) and specificity (80% *vs* 58%).

“Table 4” cross tabulates the real HCV status and the risk status based on EGCRISC old and new cutoff points. The results of the old EGCRISC show a significant association and a considerable degree of agreement with the real HCV status in each category. The highest agreement levels were mainly for males and the lowest agreement level was for females <45 years. This discrepancy in agreement levels was eliminated after the use of the new cutoff points of EGCRISC while keeping the significant association. The positive posttest probability ranged from 5% to 37.2% whereas the negative posttest probability was in the range 1% to 17%.

**Table 4:** HCV status based on standard lab and extracted scoring system of selected risk factors.

Strata	Standard		Score Categories				*P*~	(preTP) Agreement (%)	PPTP (%)	NPTP (%)	Score Categories				*P*~	(preTP) Agreement (%)	PPTP (%)	NPTP (%)
			(old cut off)								(new cut off)							
			Negative		Positive						Negative		Positive					
			N	%	N	%					N	%	N	%				
Male <45 yrs	Anti-HCV ELISA	Negative	1123	69.9	431	26.8	0.0001	72.3	8.3	1.2	1554	96.7	0	0.0	-	96.7		
		Positive	14	0.9	39	2.4					53	3.3	0	0.0				
	PCR	Negative	1126	70.1	443	27.6	0.0001	71.8	5.7	1.0	1126	70.1	443	27.6	0.001	71.8	5.7	1.0
		Positive	11	0.7	27	1.7					11	0.7	27	1.7				
Male >45 yrs	Anti-HCV ELISA	Negative	576	61.1	183	19.4	0.0001	68.1	26.5	16.9	552	58.6	207	22.0	0.002	66.0	25.3	17.0
		Positive	117	12.4	66	7.0					113	12.0	70	7.4				
	PCR	Negative	593	63	192	20.4	0.0001	69.1	23.0	14.4	568	60.3	217	23.0	0.004	66.7	21.7	14.6
		Positive	100	10.6	57	6.1					97	10.3	60	6.4				
Females <45 yrs	Anti-HCV ELISA	Negative	329	30.5	720	66.7	0.028	33.2	3.9	0.3	721	66.8	328	30.4	0.021	68.8	6.3	1.1
		Positive	1	0.1	29	2.7					8	0.7	22	2.0				
	PCR	Negative	329	30.5	729	67.6	0.026	32.4	2.7	0.3	724	67.1	334	31.0	0.034	68.6	4.6	0.69
		Positive	1	0.1	20	1.9					5	0.5	16	1.5				
Females >45 yrs	Anti-HCV ELISA	Negative	287	30.2	536	56.4	0.0001	42.7	18.2	3.0	656	69.0	167	17.6	0.0001	79.4	37.2	4.2
		Positive	9	0.9	119	12.5					29	3.0	99	10.4				
	PCR	Negative	288	30.3	549	57.7	0.0001	41.4	16.2	2.7	660	69.4	177	18.6	0.0001	78.8	33.4	3.6
		Positive	8	0.8	106	11.1					25	2.6	89	9.4				

~Exact Sig. (2-sided) McNemar test

preTP = Pre test probability (Agreement)

PPTP = Positive posttest probability

NPTP = Negative posttest probability

**Fig 2:**
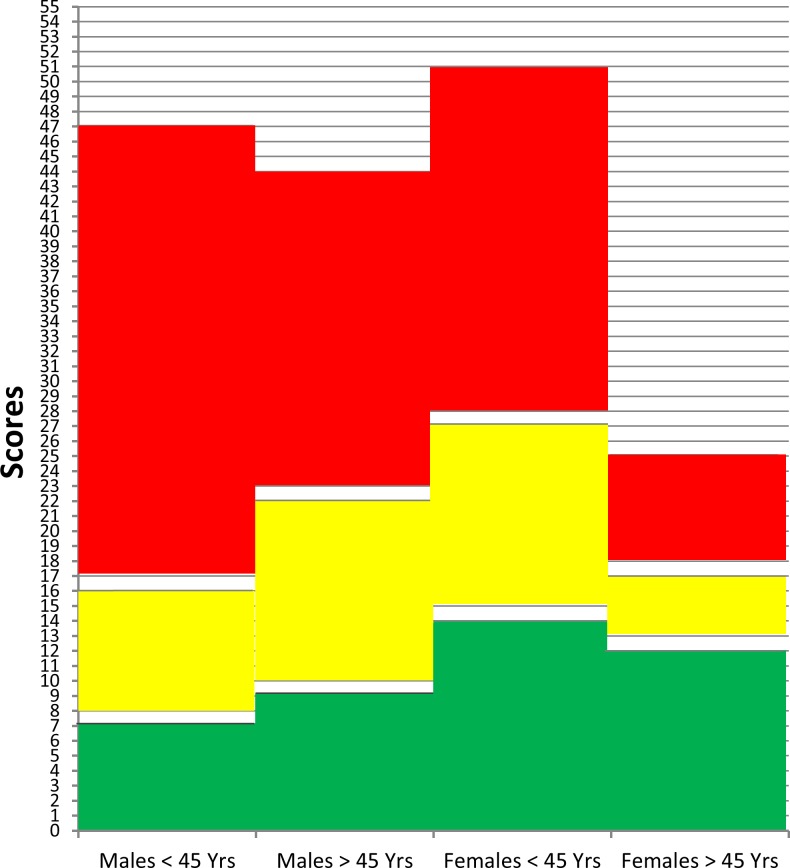
Limits for risk of having HCV based on the scoring system of the selected risk factors. The questions are not equally weighed.

For applicability we used the zones classification to rate the risk score instead of having a positive/negative test result. The limits for being at risk of having HCV based on validated scoring system of the selected risk factors are shown in (S2 Table and Fig 2). The risk is classified as low (subjects lie in the Green zone), borderline (subjects lie in Yellow zone), or high (subjects lie in Red zone).

“Table 5” highlights the zones classification and performance in the four different groups. The negative predictive value (NPV) ranged from 82% for yellow and red zones in males >45 years to 99% for younger groups. Positive predictive values (PPV) were high for red zones in older age groups compared to the younger age groups.

**Table 5:** EGCRISC zones classification and performance.

Age Category Lab. Test			Zone						Yellow Zone		Red Zone	
			Green		Yellow		Red		PPV (%)	NPV (%)	PPV (%)	NPV (%)
			No	%	No	%	No	%				
Male <45 years	Anti-HCV ELISA	Negative	598	38.5	708	45.6	248	16.0	4	99	7	99
		Positive	4	7.5	29	54.7	20	37.7				
	PCR	Negative	598	38.1	716	45.6	255	16.3	3	99	6	99
		Positive	4	10.5	21	55.3	13	34.2				
Male >45 years	Anti-HCV ELISA	Negative	644	84.8	105	13.8	10	1.3	12	82	75	82
		Positive	139	76.0	14	7.7	30	16.4				
	PCR	Negative	666	84.8	106	13.5	13	1.7	11	85	68	85
		Positive	117	74.5	13	8.3	27	17.2				
Female <45 years	Anti-HCV ELISA	Negative	348	33.2	472	45.0	229	21.8	2	99	8	99
		Positive	4	13.3	8	26.7	18	60.0				
	PCR	Negative	350	33.1	474	44.8	234	22.1	2	99	5	99
		Positive	2	9.5	6	28.6	13	61.9				
Female >45 years	Anti-HCV ELISA	Negative	493	59.9	284	34.5	46	5.6	13	97	61	97
		Positive	16	12.5	40	31.3	72	56.3				
	PCR	Negative	497	59.4	286	34.2	54	6.5	12	98	54	98
		Positive	12	10.5	38	33.3	64	56.1				

## Discussion

Although active HCV infection has an estimated national prevalence of 4% in the population age 1–59 years[14], the present study revealed a seroprevalence of 8.6% and active infection in 7.2% of those aged > 15 years. Egypt has a large reservoir of HCV and the disease transmission is ongoing [15, 16]. However, there is no adopted strategy for HCV case finding in primary health care settings in Egypt and the rates of detection remains beyond the CDC goals [17]. Spotting individuals at increased risk who should be screened for infection is a critical step. Our group has previously derived a simple risk assessment predication tool (EGCRISC) based on patient-reported yes/no questions to be used as a first-level screening tool in identifying subjects who should undergo serologic testing for HCV antibodies (phase I)[13]. In this study, we validated the proposed model in a sample depicting the Egyptian population.

Compared to the data derived from the first development phase and initial testing of EGCRISC, the probability threshold for HCV seropositivity based on our prediction model was increased except for the category male >45 years, we respected the use of the old cutoff point which had better agreement and performance. These new cutoff points will oust the need for serologic HCV antibody testing in a considerable number of uninfected subjects. The testing counsel of the proposed tool agreed well with the HCV status in this study and this features its validity, high diagnostic value, and hopefully cost-effectiveness.

A number of worldwide studies (Table 6) have developed or appraised questionnaire tools for HCV infection risk assessment [5, 18–22]. Of those that evaluated accuracy and feasibility in clinical practice, a harmony between sensitivity and specificity was respected to guarantee cogency, diagnostic performance, and cost-effectiveness of the selection method.

**Table 6:** Literature overview of the different HCV risk assessment tools.

Reference	Study population	Respondent (n)	Age (years)	HCV prevalence	Type of study	Screening tool	cut off discriminative point	Sensitivity	Specificity
Lapane et al., 1998	Top of Formdatabase of a national hepatitis screening program that included self-referred individuals screened for viral hepatitis in 40 urban hospitals in USABottom of Form	13,997		20%	cross sectional	Risk Factors based Questionnaire (Model 1)	Probability 7% in a mathematical model	65.0%	84.0%
				29%		Model 2	Individuals with any socially intrusive risk factors (history of IV drug use, history of sex with IV drug user) or socially nonintrusive risk factors (history of blood transfusion, male gender, age 30–49 yr)	69.0%	74.0%
				25%		Model 3	Individuals with two or more socially nonintrusive risk factors.	53.0%	77.0%
				12%		Model 4	Performing ALT on all, followed by EIA on those with elevated ALT	63.0%	92.0%
Nguyen et al., 2005	patients attending general medicine practice and hepatology practice at Thomas Jefferson University hospital	207 with unknown HCV status and 222 HCV +ve patients	18–60	1.5% in general medicine patients	cross sectional	a 7 item questionnaire based on variables found significantly associated with HCV infection in multivariate model of exposures	4 or more risk factors are present	24.4%	99.4%
Mallette et al., 2008	Veterans presenting for care and participated in risk stratification screening program at the Providence VA Medical Center (USA)	25,701	25–92 (mean = 58.7)	7.30%	cross sectional	a self-administered questionnaire identifying common HCV risk factors	Patients who answer yes to any of risk factors are offered anti-HCV antibody testing.		
McGinn et al., 2008	patients attending an inner-city primary care clinic	1000	55 (mean)	8.30%	cross sectional	A 27-item questionnaire assessing 5 HCV risk factor domains: work, medical, exposure, personal care, and social history. Questions were inspired from the literature and the clinical experience	1 or more positive domains	90.0%	31.0%
							3 or more positive domains	34.0%	97.0%
Zuure et al., 2010	patients with known HCV status	289	36–59	0.1%-0.4% in general Dutch population	cross sectional	core risk assessment questionnaire		85.90%	64.30%
						extended risk assessment questionnaire		89.40%	73.7%
	patients of an outpatient clinic for sexually transmitted infections (Netherland)	985	47–60			extended risk assessment questionnaire		90.0%	86.6%
Wand et al., 2012	IVDUs attended Needle and Syringe Programs in Australia	16,127	34 (mean)	51%	cross sectional and prospective cohorts	a brief self-administered questionnaire on demographic characteristics and injecting and sexual risk behaviors	>10	89.0%	16.0%
							>15	78.0%	33.0%
							>20	60.0%	54.0%
							>25	41.0%	70.0%

The present study has several strengths, among which comes the internal validation of our predictive model through internal case control and cross sectional data sets. Categorizing risk factors by age and gender enhanced the properties of our scoring tool. Also, the large representative sample of 4579 participant is ideal in risk assessment. Furthermore, the use of a proportionally allocated population based sample asserts that the derived results feature the Egyptian population. The study conducted by McGinn et al., in USA was limited by surveying population coming from an inner-city primary care practice, thus the results do not represent the true community [21]. Other studies validated their tool on hospital based small samples [19, 22] or on certain risk groups [5, 20].

Internet screening appears appropriate, attainable, and more cost-effective comparing to other strategies as it abates health care consultation costs [22]. An interactive electronic version of EGCRISC is now available at www.virus-c.com. The tool is enabled with a calculator for weighing the risk score and estimating the risk. Depending on the obtained score, the subject will be given a tailored recommendation that he/she should discuss with his/her professional healthcare provider. This instrument will empower the detection of silent chronic HCV cases in Egypt. People who are concerned about their probable HCV infection state will be boosted to assess their risk and pursue diagnosis. Filling out a risk assessment questionnaire via internet ensure anonymity and provide better chance for understanding its purpose. Individuals will therefore recall relevant information and disclose risky behaviors differently from those collected through interviewing in a survey or health care settings. However, this might result is difference in the sensitivity and specificity of our validated tool. Nevertheless, although most of individuals in Egypt can have access to internet, not all of them have sufficient literacy or mastery to employ it. According to the 2015 EHIS (Egypt Health Issues Survey)[14], 22% of women and 8% of men age 15–59 are illiterate. It also reported that around 37% of adult men and 22% of adult women use computer and internet at least once a week. These factors may represent a challenge for the wide use of our proposed web-based screening. Marketing the use of EGCRISC will be adequately advertised through mass media outlets. Egyptians are regularly exposed to mass media particularly the television (99%) that have been traditionally used to convey health messages to the population. Additionally, reports will be disseminated to health authorities recommending the wide use of EGCRISC in primary care settings. Training workshops for primary care providers are planned to be held after phase III (a governorate-based EGCRISC application phase).

Prediction questionnaire tool was proofed to be a adequate, feasible and conductive instrument, accomplishing the growing needs for the screening of several diseases including dementia [23], type 2 diabetes [24], osteoporosis [25], and HCV infection among high risk groups [5]. A composite screening tool was also developed for chronic kidney disease, cardiovascular disease and type 2 diabetes screening in the same individual [26]. Moreover, risk estimation approaches were envisaged as an alternative for the diagnosis of some diseases such as cancer and cardiovascular disorders and have been widely exploited in public health and clinical care decision-making process [6].

The perspectives of the current work are to assess the feasibility and potential shortcoming of risk-based screening using our proposed prediction tool in the clinical practice. Also, to address its cost effectiveness in HCV detection and the cost of treating early/minimal liver diseased populations. A future study is foreseen by our group to assess the cost-effectiveness of EGCRISC in internet-based and alternative programs compared with other strategies, such as mass screening or screening evidently high risk groups. The cost-effectiveness analysis should take into consideration not only abating eventual health care costs of identified HCV-infected individuals, but also expenditure associated with HCV patient who would not be detected using one of the aforementioned strategies. We also recommend further validation of our questionnaire tool particularly in countries and regions with comparable prevalence and setting to those in Egypt.

In conclusion, we have validated a simplified tool (EGCRISC) to assess HCV risk in the general population in Egypt and demonstrated its diagnostic value. EGCRISC can taper the need for mass serologic screening in those at apparently very low or no risk. Widespread use of electronic and self- or interviewer-administered risk-based screening strategy may facilitate and promote HCV screening and detection in diverse populations. Targeted HCV screening in high risk individuals is more cost-effective and can be beneficial in early identification of individuals at risk for progressive liver disease who may benefit from counseling and prompt treatment to reduce HCV-related liver injury. An impact analysis or randomized control trial of this model is warranted to evaluate both the clinical value and cost-effectiveness.

## Supporting Information

S1 Table Stratified analysis of HCV risk factors by age and gender. (DOCX)

S2 Table Limits for risk of having HCV based on the scoring system of the selected risk factors. (DOCX)
