# Supplementary material for: Validation of EGCRISC for Chronic Hepatitis C Infection Screening and Risk Assessment in the Egyptian Population
Source: PLoS One. 2016 Dec 21;11(12):e0168649. doi: 10.1371/journal.pone.0168649 (PMC5176306; doi:10.1371/journal.pone.0168649)
Supplement: S1 Table — (DOCX) [file pone.0168649.s001.docx]

S1 Table. Stratified analysis of HCV risk factors by age and gender

| **Characteristics** | | **Female** | | | | | | | | **Male** | | | | | | | |
| --- | --- | --- | --- | --- | --- | --- | --- | --- | --- | --- | --- | --- | --- | --- | --- | --- | --- |
|  |  | **<45** | | | | **45+** | | | | **<45** | | | | **45+** | | | |
|  |  | **Negative** | | **Positive** | | **Negative** | | **Positive** | | **Negative** | | **Positive** | | **Negative** | | **Positive** | |
|  |  | **No** | **%** | **No** | **%** | **No** | **%** | **No** | **%** | **No** | **%** | **No** | **%** | **No** | **%** | **No** | **%** |
| **Residence** | Urban | 368 | 35.1 | 9 | 30 | 291 | 35.4 | 15 | 11.7 | 845 | 54.4 | 24 | 45.3 | 330 | 43.5 | 39 | 21.3 |
|  | Rural | 681 | 64.9 | 21 | 70 | 532 | 64.6 | 113 | 88.3 | 709 | 45.6 | 29 | 54.7 | 429 | 56.5 | 144 | 78.7 |
|  | ***FEP*** | 0.565 | | | | **0.001** | | | | 0.191 | | | | **0.001** | | | |
| **Education** | Illiterate /read & write | 237 | 22.6 | 12 | 40 | 559 | 67.9 | 115 | 89.8 | 259 | 16.7 | 16 | 30.2 | 385 | 50.7 | 118 | 64.5 |
|  | Basic | 188 | 17.9 | 3 | 10 | 31 | 3.8 | 4 | 3.1 | 355 | 22.8 | 6 | 11.3 | 125 | 16.5 | 23 | 12.6 |
|  | Secondary | 438 | 41.8 | 14 | 46.7 | 163 | 19.8 | 7 | 5.5 | 689 | 44.3 | 22 | 41.5 | 147 | 19.4 | 29 | 15.8 |
|  | University / more | 186 | 17.7 | 1 | 3.3 | 70 | 8.5 | 2 | 1.6 | 251 | 16.2 | 9 | 17 | 102 | 13.4 | 13 | 7.1 |
|  | ***MCP*** | **0.036** | | | | **0.001** | | | | **0.035** | | | | **0.006** | | | |
| **Marital status** | Single | 138 | 13.2 | 0 | 0 | 5 | 0.6 | 0 | 0 | 314 | 20.2 | 4 | 7.5 | 8 | 1.1 | 1 | 0.5 |
|  | Married | 846 | 80.6 | 23 | 76.7 | 579 | 70.4 | 84 | 65.6 | 1229 | 79.1 | 49 | 92.5 | 740 | 97.5 | 180 | 98.4 |
|  | Divorced/widow | 65 | 6.2 | 7 | 23.3 | 239 | 29 | 44 | 34.4 | 11 | 0.7 | 0 | 0 | 11 | 1.4 | 2 | 1.1 |
|  | ***MCP*** | **0.001** | | | | 0.334 | | | | 0.059 | | | | 0.762 | | | |
| **Job nature** | Not working | 830 | 79.1 | 29 | 96.7 | 591 | 71.8 | 109 | 85.2 | 69 | 4.4 | 3 | 5.7 | 29 | 3.8 | 7 | 3.8 |
|  | Low risk | 184 | 17.5 | 1 | 3.3 | 208 | 25.3 | 9 | 7 | 1185 | 76.3 | 40 | 75.5 | 525 | 69.2 | 124 | 67.8 |
|  | High risk | 35 | 3.3 | 0 | 0 | 24 | 2.9 | 10 | 7.8 | 300 | 19.3 | 10 | 18.9 | 205 | 27 | 52 | 28.4 |
|  | ***MCP*** | 0.062 | | | | **0.001** | | | | 0.914 | | | | 0.928 | | | |
| **Tarter injections for Bilharziasis** | No | 1020 | 97.5 | 26 | 2.5 | 650 | 91.5 | 60 | 8.5 | 1464 | 97.5 | 38 | 2.5 | 500 | 90.4 | 53 | 9.6 |
|  | Yes | 29 | 87.9 | 4 | 12.1 | 173 | 71.8 | 68 | 28.2 | 90 | 85.7 | 15 | 14.3 | 259 | 66.6 | 130 | 33.4 |
|  | ***FEP*** | **0.001** | | | | **0.001** | | | | **0.001** | | | | **0.001** | | | |
| **History of oral ulcers** | No | 645 | 96.8 | 21 | 3.2 | 502 | 89.5 | 59 | 10.5 | 1083 | 96.4 | 40 | 3.6 | 519 | 81.3 | 119 | 18.7 |
|  | Yes | 404 | 97.8 | 9 | 2.2 | 321 | 82.3 | 69 | 17.7 | 471 | 97.3 | 13 | 2.7 | 240 | 78.9 | 64 | 21.1 |
|  | ***FEP*** | 0.344 | | | | **0.001** | | | | 0.367 | | | | 0.384 | | | |
| **Acupuncture** | No | 679 | 97.6 | 17 | 2.4 | 543 | 86.2 | 87 | 13.8 | 1390 | 96.9 | 44 | 3.1 | 534 | 82.5 | 113 | 17.5 |
|  | Yes | 370 | 96.6 | 13 | 3.4 | 280 | 87.2 | 41 | 12.8 | 164 | 94.8 | 9 | 5.2 | 225 | 76.3 | 70 | 23.7 |
|  | ***FEP*** | 0.363 | | | | 0.658 | | | | 0.138 | | | | **0.024** | | | |
| **Invasive procedure** | No | 1006 | 97.1 | 30 | 2.9 | 786 | 86.2 | 126 | 13.8 | 1532 | 96.7 | 53 | 3.3 | 751 | 80.7 | 180 | 19.3 |
|  | Yes | 43 | 100 | 0 | 0 | 37 | 94.9 | 2 | 5.1 | 22 | 100 | 0 | 0 | 8 | 72.7 | 3 | 27.3 |
|  | ***FEP*** | 0.258 | | | | 0.12 | | | | 0.383 | | | | 0.508 | | | |
| **History of incarceration** | No | 1049 | 97.2 | 30 | 2.8 | 822 | 86.5 | 128 | 13.5 | 1516 | 96.7 | 52 | 3.3 | 711 | 80.2 | 175 | 19.8 |
|  | Yes | 0 | 0 | 0 | 0 | 1 | 100 | 0 | 0 | 38 | 97.4 | 1 | 2.6 | 48 | 85.7 | 8 | 14.3 |
|  | ***FEP*** | - | | | | 0.936 | | | | 0.795 | | | | 0.316 | | | |
| **Opioid injection** | No | 1048 | 97.2 | 30 | 2.8 | 823 | 86.5 | 128 | 13.5 | 1553 | 96.8 | 52 | 3.2 | 759 | 80.6 | 183 | 19.4 |
|  | Yes | 1 | 100 | 0 | 0 | 0 | 0 | 0 | 0 | 1 | 50 | 1 | 50 | 0 | 0 | 0 | 0 |
|  | ***FEP*** | 0.866 | | | | - | | | | **0.001** | | | | - | | | |
| **Drink alcohol** | No | 1041 | 97.2 | 30 | 2.8 | 818 | 86.5 | 128 | 13.5 | 1427 | 96.9 | 46 | 3.1 | 712 | 81.3 | 164 | 18.7 |
|  | Yes | 8 | 100 | 0 | 0 | 5 | 100 | 0 | 0 | 127 | 94.8 | 7 | 5.2 | 47 | 71.2 | 19 | 28.8 |
|  | ***FEP*** | 0.631 | | | | 0.377 | | | | 0.192 | | | | **0.046** | | | |
| **Shisha smoking** | No | 1016 | 97.1 | 30 | 2.9 | 730 | 85.8 | 121 | 14.2 | 1130 | 96.4 | 42 | 3.6 | 547 | 82.3 | 118 | 17.7 |
|  | Yes | 33 | 100 | 0 | 0 | 93 | 93 | 7 | 7 | 424 | 97.5 | 11 | 2.5 | 212 | 76.5 | 65 | 23.5 |
|  | ***FEP*** | 0.324 | | | | **0.045** | | | | 0.293 | | | | **0.043** | | | |
| **Previous stay at camps/hostels** | No | 983 | 97.1 | 29 | 2.9 | 807 | 86.6 | 125 | 13.4 | 825 | 96.8 | 27 | 3.2 | 265 | 80.1 | 66 | 19.9 |
|  | Yes | 66 | 98.5 | 1 | 1.5 | 16 | 84.2 | 3 | 15.8 | 729 | 96.6 | 26 | 3.4 | 494 | 80.9 | 117 | 19.1 |
|  | ***FEP*** | 0.508 | | | | 0.764 | | | | 0.758 | | | | 0.77 | | | |
| **Partner with sexual diseases** | No | 1016 | 97.1 | 30 | 2.9 | 802 | 86.3 | 127 | 13.7 | 1474 | 96.6 | 52 | 3.4 | 729 | 80.9 | 172 | 19.1 |
|  | Yes | 33 | 100 | 0 | 0 | 21 | 95.5 | 1 | 4.5 | 80 | 98.8 | 1 | 1.2 | 30 | 73.2 | 11 | 26.8 |
|  | ***FEP*** | 0.324 | | | | 0.215 | | | | 0.286 | | | | 0.221 | | | |
| **Partner infected with HCV** | No | 1013 | 97.4 | 27 | 2.6 | 753 | 86.7 | 116 | 13.3 | 1544 | 96.7 | 52 | 3.3 | 740 | 80.8 | 176 | 19.2 |
|  | Yes | 36 | 92.3 | 3 | 7.7 | 70 | 85.4 | 12 | 14.6 | 10 | 90.9 | 1 | 9.1 | 19 | 73.1 | 7 | 26.9 |
|  | ***FEP*** | 0.057 | | | | 0.744 | | | | 0.28 | | | | 0.327 | | | |
| **Un explained fatigue during last 6 months** | No | 662 | 97.4 | 18 | 2.6 | 460 | 90.2 | 50 | 9.8 | 1224 | 96.8 | 40 | 3.2 | 543 | 84.6 | 99 | 15.4 |
|  | Yes | 387 | 97 | 12 | 3 | 363 | 82.3 | 78 | 17.7 | 330 | 96.2 | 13 | 3.8 | 216 | 72 | 84 | 28 |
|  | ***FEP*** | 0.728 | | | | **0.001** | | | | 0.565 | | | | **0.001** | | | |
| **Working abroad previously** | No | 1006 | 97.2 | 29 | 2.8 | 764 | 86 | 124 | 14 | 1343 | 96.8 | 45 | 3.2 | 502 | 80.7 | 120 | 19.3 |
|  | Yes | 43 | 97.7 | 1 | 2.3 | 59 | 93.7 | 4 | 6.3 | 211 | 96.3 | 8 | 3.7 | 257 | 80.3 | 63 | 19.7 |
|  | ***FEP*** | 0.834 | | | | 0.087 | | | | 0.752 | | | | 0.885 | | | |
| **Travel place** | No | 1005 | 97.2 | 29 | 2.8 | 763 | 86 | 124 | 14 | 1342 | 96.8 | 45 | 3.2 | 499 | 80.6 | 120 | 19.4 |
|  | Asia | 1 | 100 | 0 | 0 | 0 | 0 | 0 | 0 | 1 | 100 | 0 | 0 | 0 | 0 | 0 | 0 |
|  | Western | 3 | 100 | 0 | 0 | 7 | 100 | 0 | 0 | 16 | 100 | 0 | 0 | 14 | 82.4 | 3 | 17.6 |
|  | Arab country | 40 | 97.6 | 1 | 2.4 | 53 | 94.6 | 3 | 5.4 | 194 | 96 | 8 | 4 | 244 | 80.3 | 60 | 19.7 |
|  | Africa | 0 | 0 | 0 | 0 | 0 | 0 | 1 | 100 | 1 | 100 | 0 | 0 | 2 | 100 | 0 | 0 |
|  | ***MCP*** | 0.987 | | | | **0.012** | | | | 0.924 | | | | 0.911 | | | |
| **Tattooing** | No | 1000 | 97.2 | 29 | 2.8 | 701 | 85.6 | 118 | 14.4 | 1477 | 96.9 | 48 | 3.1 | 700 | 80 | 175 | 20 |
|  | Yes | 49 | 98 | 1 | 2 | 122 | 92.4 | 10 | 7.6 | 77 | 93.9 | 5 | 6.1 | 59 | 88.1 | 8 | 11.9 |
|  | ***FEP*** | 0.731 | | | | **0.033** | | | | 0.145 | | | | 0.108 | | | |
| **Ear / body piercing** | No | 17 | 94.4 | 1 | 5.6 | 12 | 80 | 3 | 20 | 1539 | 96.7 | 52 | 3.3 | 740 | 80.6 | 178 | 19.4 |
|  | Yes | 1032 | 97.3 | 29 | 2.7 | 811 | 86.6 | 125 | 13.4 | 15 | 93.8 | 1 | 6.3 | 19 | 79.2 | 5 | 20.8 |
|  | ***FEP*** | 0.47 | | | | 0.454 | | | | 0.506 | | | | 0.86 | | | |
| **Shared instruments** | No | 139 | 96.5 | 5 | 3.5 | 163 | 86.2 | 26 | 13.8 | 401 | 95.5 | 19 | 4.5 | 201 | 79.8 | 51 | 20.2 |
|  | Yes | 910 | 97.3 | 25 | 2.7 | 660 | 86.6 | 102 | 13.4 | 1153 | 97.1 | 34 | 2.9 | 558 | 80.9 | 132 | 19.1 |
|  | ***FEP*** | 0.587 | | | | 0.894 | | | | 0.102 | | | | 0.704 | | | |
| **Use barber tools** | No | 287 | 96 | 12 | 4 | 522 | 82.9 | 108 | 17.1 | 142 | 96.6 | 5 | 3.4 | 75 | 89.3 | 9 | 10.7 |
|  | Yes | 762 | 97.7 | 18 | 2.3 | 301 | 93.8 | 20 | 6.2 | 1412 | 96.7 | 48 | 3.3 | 684 | 79.7 | 174 | 20.3 |
|  | ***FEP*** | 0.127 | | | | **0.001** | | | | 0.941 | | | | **0.034** | | | |
| **Pierced with blood contaminated object** | No | 925 | 97.3 | 26 | 2.7 | 769 | 86.8 | 117 | 13.2 | 1440 | 96.7 | 49 | 3.3 | 717 | 81.3 | 165 | 18.7 |
|  | Yes | 124 | 96.9 | 4 | 3.1 | 54 | 83.1 | 11 | 16.9 | 114 | 96.6 | 4 | 3.4 | 42 | 70 | 18 | 30 |
|  | ***FEP*** | 0.801 | | | | 0.397 | | | | 0.954 | | | | **0.032** | | | |
| **Bitten by animal** | No | 858 | 97.2 | 25 | 2.8 | 679 | 86.6 | 105 | 13.4 | 1230 | 96.7 | 42 | 3.3 | 619 | 81 | 145 | 19 |
|  | Yes | 191 | 97.4 | 5 | 2.6 | 144 | 86.2 | 23 | 13.8 | 324 | 96.7 | 11 | 3.3 | 140 | 78.7 | 38 | 21.3 |
|  | ***FEP*** | 0.829 | | | | 0.896 | | | | 0.987 | | | | 0.472 | | | |
| **Exposed to blood** | No | 781 | 97.3 | 22 | 2.7 | 703 | 87.8 | 98 | 12.2 | 1183 | 96.4 | 44 | 3.6 | 638 | 81.9 | 141 | 18.1 |
|  | Yes | 268 | 97.1 | 8 | 2.9 | 120 | 80 | 30 | 20 | 371 | 97.6 | 9 | 2.4 | 121 | 74.2 | 42 | 25.8 |
|  | ***FEP*** | 0.89 | | | | **0.011** | | | | 0.246 | | | | **0.024** | | | |
| **Blood / blood products transfusion** | No | 949 | 98.1 | 18 | 1.9 | 720 | 87.3 | 105 | 12.7 | 1493 | 97.1 | 44 | 2.9 | 696 | 81.4 | 159 | 18.6 |
|  | Yes | 100 | 89.3 | 12 | 10.7 | 103 | 81.7 | 23 | 18.3 | 61 | 87.1 | 9 | 12.9 | 63 | 72.4 | 24 | 27.6 |
|  | ***FEP*** | **0.001** | | | | **0.09** | | | | **0.001** | | | | **0.043** | | | |
| **History of jaundice** | No | 971 | 97.3 | 27 | 2.7 | 760 | 86.7 | 117 | 13.3 | 1418 | 96.6 | 50 | 3.4 | 665 | 80.7 | 159 | 19.3 |
|  | Yes | 78 | 96.3 | 3 | 3.7 | 63 | 85.1 | 11 | 14.9 | 136 | 97.8 | 3 | 2.2 | 94 | 79.7 | 24 | 20.3 |
|  | ***FEP*** | 0.599 | | | | 0.712 | | | | 0.431 | | | | 0.789 | | | |
| **Family member with hepatic disease** | No | 839 | 97.3 | 23 | 2.7 | 732 | 87.5 | 105 | 12.5 | 1265 | 96.6 | 44 | 3.4 | 667 | 81.2 | 154 | 18.8 |
|  | Yes | 210 | 96.8 | 7 | 3.2 | 91 | 79.8 | 23 | 20.2 | 289 | 97 | 9 | 3 | 92 | 76 | 29 | 24 |
|  | ***FEP*** | 0.655 | | | | **0.025** | | | | 0.766 | | | | 0.176 | | | |
| **Taken blood sample** | No | 86 | 98.9 | 1 | 1.1 | 88 | 88.9 | 11 | 11.1 | 294 | 97.7 | 7 | 2.3 | 123 | 83.7 | 24 | 16.3 |
|  | Less than 10 years | 730 | 97.5 | 19 | 2.5 | 479 | 86 | 78 | 14 | 1075 | 97.3 | 30 | 2.7 | 473 | 81.6 | 107 | 18.4 |
|  | More than 10 years | 233 | 95.9 | 10 | 4.1 | 256 | 86.8 | 39 | 13.2 | 185 | 92 | 16 | 8 | 163 | 75.8 | 52 | 24.2 |
|  | ***MCP*** | 0.269 | | | | 0.732 | | | | **0.001** | | | | 0.113 | | | |
| **Previous hospitalization** | No | 415 | 97.2 | 12 | 2.8 | 327 | 84.9 | 58 | 15.1 | 873 | 96.4 | 33 | 3.6 | 299 | 78.5 | 82 | 21.5 |
|  | Yes | 634 | 97.2 | 18 | 2.8 | 496 | 87.6 | 70 | 12.4 | 681 | 97.1 | 20 | 2.9 | 460 | 82 | 101 | 18 |
|  | ***FEP*** | 0.961 | | | | 0.323 | | | | 0.38 | | | | 0.18 | | | |
| **Bilharziasis** | No | 869 | 98.4 | 14 | 1.6 | 555 | 93.1 | 41 | 6.9 | 1050 | 97.6 | 26 | 2.4 | 412 | 92.2 | 35 | 7.8 |
|  | Yes | 180 | 91.8 | 16 | 8.2 | 268 | 75.5 | 87 | 24.5 | 504 | 94.9 | 27 | 5.1 | 347 | 70.1 | 148 | 29.9 |
|  | ***FEP*** | **0.001** | | | | **0.001** | | | | **0.005** | | | | **0.001** | | | |
| **Genital** **ulcers** | No | 721 | 98 | 15 | 2 | 586 | 87.1 | 87 | 12.9 | 1334 | 96.5 | 48 | 3.5 | 595 | 82 | 131 | 18 |
|  | Yes | 328 | 95.6 | 15 | 4.4 | 237 | 85.3 | 41 | 14.7 | 220 | 97.8 | 5 | 2.2 | 164 | 75.9 | 52 | 24.1 |
|  | ***FEP*** | **0.030** | | | | 0.454 | | | | 0.33 | | | | **0.049** | | | |
| **Circumcision** | No | 271 | 98.2 | 5 | 1.8 | 76 | 93.8 | 5 | 6.2 | 564 | 97.2 | 16 | 2.8 | 150 | 93.2 | 11 | 6.8 |
|  | Yes | 778 | 96.9 | 25 | 3.1 | 747 | 85.9 | 123 | 14.1 | 990 | 96.4 | 37 | 3.6 | 609 | 78 | 172 | 22 |
|  | ***FEP*** | 0.256 | | | | **0.045** | | | | 0.363 | | | | **0.001** | | | |
