# Supplementary material for: Validation of EGCRISC for Chronic Hepatitis C Infection Screening and Risk Assessment in the Egyptian Population
Source: PLoS One. 2016 Dec 21;11(12):e0168649. doi: 10.1371/journal.pone.0168649 (PMC5176306; doi:10.1371/journal.pone.0168649)
Supplement: S2 Table — (DOCX) [file pone.0168649.s002.docx]

S2 Table. Limits for risk of having HCV based on the scoring system of the selected risk factors

| Zones | Total  score | Cluster Number of Case | | |
| --- | --- | --- | --- | --- |
|  |  | Green zone | Yellow zone | Red zone |
| Males < 45 Yrs | Minimum | 0 | 8 | 17 |
|  | Maximum | 7 | 16 | 47 |
|  | Mean | 3.29 | 11.62 | 20.56 |
| Males > 45 Yrs | Minimum | 0 | 10 | 23 |
|  | Maximum | 9 | 22 | 44 |
|  | Mean | 4.23 | 10.84 | 37.75 |
| Females < 45 Yrs | Minimum | 0 | 15 | 28 |
|  | Maximum | 14 | 27 | 51 |
|  | Mean | 5.82 | 22.14 | 32.49 |
| Females > 45 Yrs | Minimum | 0 | 13 | 18 |
|  | Maximum | 12 | 17 | 25 |
|  | Mean | 6.91 | 15.28 | 20.31 |
